# Stroke profile and care during the COVID-19 pandemic: What changed and what did not? A prospective cohort from Joinville, Brazil

**DOI:** 10.3389/fneur.2023.1122875

**Published:** 2023-02-16

**Authors:** Felipe Ibiapina dos Reis, Pedro Silva Correa de Magalhães, Henrique Diegoli, Alexandre Luiz Longo, Carla Heloisa Cabral Moro, Juliana Antunes Safanelli, Vivian Nagel, Marcos Christiano Lange, Viviane Flumignan Zétola

**Affiliations:** ^1^Division of Neurology, University of Joinville's Region, Joinville, Santa Catarina, Brazil; ^2^Division of Neurology, Hospital Municipal São José, Joinville, Santa Catarina, Brazil; ^3^Hospital Municipal São José, Joinville Stroke Registry, Joinville, Santa Catarina, Brazil; ^4^Federal University of Paraná, Hospital de Clínicas, Curitiba, Paraná, Brazil

**Keywords:** stroke care, stroke profile, epidemiology, mortality, incidence, COVID-19, coronavirus

## Abstract

**Introduction:**

The COVID-19 pandemic has wrought negative consequences concerning quality of care for stroke patients since its onset. Prospective population-based data about stroke care in the pandemic are limited. This study aims to investigate the impact of COVID-19 pandemic on stroke profile and care in Joinville, Brazil.

**Methods:**

A prospective population-based cohort enrolled the first-ever cerebrovascular events in Joinville, Brazil, and a comparative analyzes was conducted between the first 12 months following COVID-19 restrictions (starting March 2020) and the 12 months just before. Patients with transient ischemic attack (TIA) or stroke had their profiles, incidences, subtypes, severity, access to reperfusion therapy, in-hospital stay, complementary investigation, and mortality compared.

**Results:**

The profiles of TIA/stroke patients in both periods were similar, with no differences in gender, age, severity, or comorbidities. There was a reduction in incidence of TIA (32.8%; *p* = 0.003). In both periods, intravenous thrombolysis (IV) and mechanical thrombectomy (MT) rates and intervals from door to IV/MT were similar. Patients with cardioembolic stroke and atrial fibrillation had their in-hospital stay abbreviated. The etiologic investigation was similar before and during the pandemic, but there were increases in cranial tomographies (*p* = 0.02), transthoracic echocardiograms (*p* = 0.001), chest X-rays (*p* < 0.001) and transcranial Doppler ultrasounds (*p* < 0.001). The number of cranial magnetic resonance imaging decreased in the pandemic. In-hospital mortality did not change.

**Discussion:**

The COVID-19 pandemic is associated with a reduction in TIA, without any influence on stroke profile, the quality of stroke care, in-hospital investigation or mortality. Our findings show an effective response by the local stroke care system and offer convincing evidence that interdisciplinary efforts are the ideal approach to avoiding the COVID-19 pandemic's negative effects, even with scarce resources.

## 1. Introduction

Since the early spread of the novel coronavirus SARS-CoV-2 around the world, many doubts have arisen about the consequences of the pandemic, including SARS-CoV-2 itself and its association with neurological complications, especially cerebrovascular diseases. After early reporting of severe stroke in young infected patients, an association between COVID-19 and stroke was suggested and quickly generated concern (1–6).

In addition to the potential direct vascular damage attributed to the new coronavirus, the very dynamics of the pandemic and the increasing number of COVID-19 patients overloading hospitals have forced several countries to remodel their health services in view of imminent collapse (7). The attention of public authorities and most health resources were quickly directed to manage the pandemic, putting even greater strain on stroke-dedicated services (8). Health professionals were removed from their posts, some infected and others because of being at higher risk; human resources and equipment were directed to infected patients; non-specialized teams became in charge of treating severe mimicking diseases, including meningitis, other infections, cardiovascular disease and stroke. The general population, influenced by stay-at-home requirements and afraid of contracting COVID-19, grew more reluctant to seeking medical assistance. Access to outpatient clinics became restricted and the control of vascular risk factors was affected. All these aspects were called “the collateral effect of the pandemic,” and have negatively influenced the hospitalization rates, time-sensitive reperfusion therapies, pathology severity, and quality of care for stroke and other potentially lethal diseases (9, 10).

Observational studies showed an 11.5–41.4% reduction of all stroke hospital admissions, in particular transient ischemic attack (TIA) and minor strokes, in the first months of the pandemic (11–16). Other studies have also revealed a significant reduction in neuroimaging for acute stroke by 22.8–39% (9, 14) and reperfusion therapies by 12.7–27% (16, 17). While observational data have clearly made inroads into our understanding of associations between COVID-19 and TIA/strokes, real-world prospective longitudinal population-based data are still lacking. Such information would be especially valuable to gain further understanding of the nature of that possible association and, importantly, the development of clinical strategies to address it.

Our study aimed to analyze the effects of COVID-19 on the incidence of TIA/stroke, including the epidemiological profile and severity of these patients, and the response of the local health care system, in terms of stroke management, access to reperfusion therapies, in-hospital investigation, and mortality, 1 year after the pandemic outbreak. We hypothesized that stroke patient profiles would worsen over the pandemic in terms of comorbidities and pathology severity, as well as stroke admissions would decrease due to the strain the pandemic would exert on the healthcare system. It would be reasonable to expect a decline in IV and MT rates and an increase in the intervals between door and IV/MT. Finally, inadequate stroke management throughout the pandemic would result in deteriorating investigation, shortened hospital stays in order to give priority to COVID-19 patients, and unsatisfactory outcomes, including increased in-hospital mortality.

## 2. Material and methods

### 2.1. Study design

This was a prospective population-based cohort evaluating all patients with first-ever cerebrovascular events (TIA and/or stroke) in Joinville, Brazil, from March 1, 2019 to February 28, 2021. These data are a subset of the prospective local database sponsored by Municipal Law from June 12, 2013 (the Joinville Stroke Registry “JOINVASC”). Since 2005, it has registered more than 9,000 patients with TIA and stroke who had been admitted to various healthcare centers throughout the city. Data on the incidence of stroke in Joinville in 1995, 2005 to 2006, and 2012 to 2013 have been previously published (18, 19).

The study methodology was based on directives from Sudlow and Warlow (20), and the WHO (21), consisting of a step-by-step protocol procedure in the surveillance of stroke with three “s*teps,”* ensuring coverage for all in-hospital and community cases of TIA and stroke, and all deaths by stroke as well. The diagnoses were identified by their related ICD-10 codes (primary, secondary, or tertiary discharge codes) and/or classifications in stroke databases at participating centers. Both diagnosis and etiology of TIA/stroke were reviewed weekly by a stroke team, composed of neurologists and specialized nurses.

### 2.2. Setting and participants

With a population of 604,708, Joinville is the largest city in Santa Catarina State, which is located in southern Brazil (22). It is supported by three public plus three private hospitals and they all run 24-h Computed Tomography. Each one of them took part in our study. The largest hospital in the city is a public institution (Hospital Municipal São José) and the referral service for stroke care, responsible for treating eighty percent (80%) of all stroke patients. Hospital Municipal and two private hospitals regularly perform mechanical thrombectomy (MT) and intravenous thrombolysis (IV) in Joinville.

According to official data, Joinville had the highest number of COVID-19 cases and linked deaths when compared to other cities in the state (23, 24). We began collecting data in March 2020, once the first COVID-19 case was confirmed and social restrictions started (23). Inclusion criteria were (1) residency in Joinville; and (2) first-ever cerebrovascular event (TIA, ischemic stroke, intracerebral hemorrhage and subarachnoid hemorrhage) from March 1, 2019 to February 28, 2021. Exclusion criteria included (1) previous stroke or previous TIA (recurrent cases); (2) non-spontaneous intracranial hemorrhages (secondary to post-traumatic injuries); and (3) death in the first 24 h from the event, with no hospital or medical records (20).

### 2.3. Study variables and outcomes measures

All cases of first-ever TIA/Stroke from March 2019 to February 2020 were recorded, as well as from March 2020 to February 2021. These two groups were compared (12 months before pandemic or “pre-COVID” vs. 12 months after pandemic onset or “post-COVID”).

TOAST classification was used for the etiological classification of stroke (25). Bamford's Classification and the National Institute of Health Stroke Scale (NIHSS) score were used for stroke severity (26, 27). The modified Rankin Scale (mRS) was used to assess the patient's level of disability (28).

The analyzed variables were age (years old), gender (male or female), body mass index (kg/m^2^), the prevalence of tobacco use, alcohol abuse, sedentary lifestyle (physical inactivity), and pre-existing comorbidities (referred to the use of medication for more than 1 year), including hypertension, diabetes, dyslipidemia, heart diseases such as atrial fibrillation (AF), acute myocardial infarction and congestive heart failure. Measures included the final TOAST classification, Bamford's classification; NIHSS score on admission, and mRS score on admission and discharge. Finally, hospital-specific variables included reperfusion therapy rates (IV and MT), time intervals (in minutes) between admission and neuroimaging (door-to-imaging), admission and reperfusion therapies (door-to-needle for IV and door-to-puncture for MT), in-hospital stay (in days), number of performed complementary tests for in-hospital investigation, and in-hospital mortality.

The in-hospital investigation included cranial computed tomography (CT), electrocardiogram (ECG) and chest X-ray (X-ray). In cases of TIA and stabilized cerebral infarction, the sequence of tests included carotid and vertebral artery duplex (CVD), transthoracic echocardiogram (TTE) and transcranial Doppler ultrasound (TCD). According to the neurologist in charge, when clinical data suggest a cardioembolic source or heart disease, patients underwent transesophageal echocardiography (TEE) and holter cardiogram (HOLTER). The same personal judgment justifies the additional investigation with brain magnetic resonance imaging (MRI), neck and cranial magnetic resonance angiography (MRA) or computer tomography angiography (CTA). COVID-19 testing was performed for any symptomatic patient, either on hospital admission or at any time during hospitalization. Fever, cough, sore throat, stuffy or runny nose, shortness of breath, fatigue, muscle or body aches, nausea, vomiting, or diarrhea, new or unexplained loss of taste or smell were the most common symptoms to justify COVID-19 testing.

### 2.4. Statistical analysis

A frequency distribution table was set up for all variables, using mean and standard deviation for parametric variables, and median and interquartile intervals for non-parametric variables. The comparison between means for continuous variables was performed using Student's *t*-test, with a 95% confidence interval. Comparative univariate analysis between events was performed using Fisher's exact test for discrete/binary variables. Statistical analyses were carried out using Microsoft Excel^®^2010, and SPSS V18 (SPSS Inc., Chicago, IL). A *p*-value < 0.05 was considered statistically significant.

### 2.5. Funding and ethics

This was an investigator-initiated project supported by University of Joinville's Region (Univille) and the Joinville Municipal Health Department. The funders had no role in study design, data collection, data analysis, data interpretation, or writing of the report. The corresponding author had full access to all data in the study and had final responsibility for the decision to submit for publication.

The first author wrote the first draft of the manuscript with subsequent input of all co-authors. The institutional review boards from the coordinating sites (University of Joinville's Region and Federal University of Paraná) considered that the investigators did not have access to protected health information, and thus no IRB oversight was required since the study did not meet the federal description of human subject research. All patients or their legally authorized representatives were interviewed for demographic and clinical data collection, after informed written consent and local ethic committee approval.

## 3. Results

**Figure 1:**
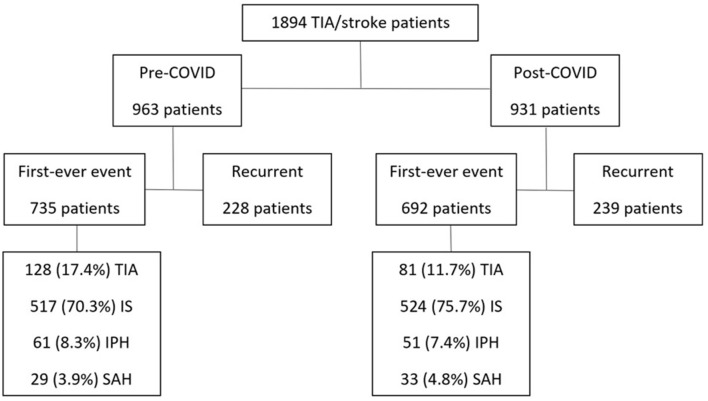
Flowchart of all TIA/stroke patients from March 2019 to February 2021. TIA, transient ischemic attack, IS, ischemic stroke; IPH, intraparenchymal hemorrhage; SAH, subarachnoid hemorrhage.

A total of 1,894 patients with cerebrovascular events were registered. 963 cases were registered before (“pre-COVID”) and 931 cases after the pandemic onset (“post-COVID”). From these 1,894 patients, 467 (228 pre-COVID plus 239 post-COVID) were defined as recurrent events and excluded from the study. A total of 735 pre-COVID plus 692 post-COVID patients have had first-ever cerebrovascular events (Figure 1). The cumulative annual crude incidences of TIA/stroke in Joinville before and after the pandemic outbreak were similar, with 124.5 and 115.8 new cases per 100,000 inhabitants, respectively (*p* = 0.171). Only 35 TIA/stroke patients tested positive for COVID-19, which represents an incidence of 5.06%.

Comparing pre-COVID and post-COVID groups, there were no differences in gender, age, body mass index (BMI) or prevalence of comorbidities (Table 1). For stroke incidences, there was a significant reduction in the number of TIA by 32.8% (*p* = 0.003), and no significant difference was observed in other stroke subtypes (Table 1). There were no differences in the NIHSS score, Bamford's Classification and higher-scored mRS 4 and 5 distributions (Table 1).

**Table 1:** Baseline information, clinical profiles, incidences, severity, access to reperfusion therapy, complementary tests, in-hospital stay, and functional outcome of first-ever TIA/stroke patients pre and post-COVID 19.

	**Pre-COVID**		**Post-COVID**		***P*-value**
	**(*****n*** = **735)**		**(*****n*** = **692)**		
Male patients, *n* (%)	366	(49.8)	377	(54.5)	0.080
Mean age, years (SD)	65.6	(14.4)	65.6	(13.9)	0.823
Patients younger than 56yo, *n* (%)	164	(22.3)	153	(22.1)	0.949
Patients younger than 46yo, *n* (%)	65	(8.8)	54	(7.8)	0.503
Body mass index, kg/m^2^ (SD)	27.1	(5.1)	27.6	(5.4)	0.084
Hypertension, *n* (%)	522	(71)	469	(67.8)	0.187
Diabetes, *n* (%)	226	(30.7)	206	(29.8)	0.729
Tobacco use, *n* (%)	142	(19.3)	151	(21.8)	0.265
Alcohol abuse, *n* (%)	47	(6.4)	53	(7.7)	0.353
Dyslipidemia, *n* (%)	243	(33.1)	196	(28.3)	0.058
Cardiopathy, *n* (%)	215	(29.3)	195	(28.2)	0.682
Atrial Fibrillation, *n* (%)	52	(7.1)	41	(5.9)	0.393
Physical inactivity, *n* (%)	483	(65.7)	485	(70.1)	0.079
**Type of stroke (final diagnosis)**, ***n*** **(%)**					
Transient ischemic attack	128	(17.4)	81	(11.7)	0.003
Subarachnoid hemorrhage	29	(3.9)	33	(4.8)	0.516
Intraparenchymal hemorrhage	61	(8.3)	51	(7.4)	0.555
Cerebral infarction	517	(70.3)	524	(75.7)	
Cardioembolic stroke (CS)	106	(14.4)	89	(12.9)	0.398
CS with AF	63	(8.6)	52	(7.5)	0.496
CS without AF	43	(5.9)	37	(5.3)	0.730
Large-artery atherosclerosis	73	(9.9)	90	(13)	0.080
Small-vessel occlusion	98	(13.3)	110	(15.9)	0.177
Other determined etiology	19	(2.6)	18	(2.6)	1.000
Undetermined etiology	221	(30.1)	217	(31.4)	0.606
Two or more causes identified	7	(1)	13	(1.9)	0.177
Negative evaluation	149	(20.3)	161	(23.3)	0.178
Incomplete evaluation	65	(8.8)	43	(6.2)	0.071
**Bamford's classification**, ***n*** **(%)**					
PACS	196	(26.7)	214	(30.9)	0.079
POCS	113	(15.4)	99	(14.3)	0.602
LACS	155	(21.1)	140	(20.2)	0.695
TACS	55	(7.5)	62	(9)	0.335
**Stroke severity**					
NIHSS 0–4, *n* (%)	339	(46.1)	351	(50.7)	0.090
NIHSS 5–8, *n* (%)	90	(12.2)	76	(11)	0.509
NIHSS >8, *n* (%)	157	(21.4)	159	(23)	0.483
mRS 4–5 (admission), *n* (%)	210	(28.6)	203	(29.3)	0.433
**Reperfusion therapies for ischemic stroke**					
Procedures, *n* (%)	65	(12.6)	76	(14.5)	0.367
Endovenous thrombolysis alone	44	(8.5)	45	(8.6)	1.000
Endovascular thrombectomy alone	12	(2.3)	20	(3.8)	0.209
Thrombolysis + thrombectomy	9	(1.7)	11	(2.1)	0.822
DTI, min (IQR)	25	(16,37)	25	(18,33)	0.960
DTN/P, min (IQR)	75	(44;106.5)	71	(49;99)	0.969
**Stroke investigation**					
Computed tomography, *n* (%)	681	(92.7)	661	(95.5)	0.025
Holter cardiogram, *n* (%)	214	(29.1)	193	(27.9)	1.000
Transthoracic echocardiogram, *n* (%)	524	(71.3)	545	(78.8)	0.001
Transesophageal echocardiogram, *n* (%)	21	(2.9)	15	(2.2)	0.500
Carotid and vertebral artery duplex, *n* (%)	580	(78.9)	535	(77.3)	0.481
Transcranial doppler ultrasound, *n* (%)	114	(15.5)	240	(34.7)	< 0.001
Chest X-ray, *n* (%)	524	(71.3)	563	(81.4)	< 0.001
Magnetic resonance imaging, *n* (%)	229	(31.2)	175	(25.3)	0.016
Magnetic resonance angiography, *n* (%)	195	(26.5)	195	(28.2)	0.513
Computed tomographic angiography, *n* (%)	20	(2.7)	26	(3.8)	0.296
Cerebral angiography, *n* (%)	64	(8.7)	66	(9.5)	0.646
**Mean in-hospital stay, days (SD)**					
Transient ischemic attack	5.9	(3.8)	6	(3.9)	0.875
Subarachnoid hemorrhage	12.2	(11.1)	19.9	(23)	0.093
Intraparenchymal hemorrhage	15.2	(19.4)	11.5	(11.7)	0.220
Cerebral infarction	10.7	(10.8)	9.3	(10)	0.029
Cardioembolic stroke (CS)	13.2	(13.9)	9.4	(7.8)	0.019
CS with AF	11.9	(13.4)	7.4	(6.6)	0.021
CS without AF	15.1	(14.7)	12.3	(8.6)	0.290
Large-artery atherosclerosis	12.7	(9.1)	11	(11.7)	0.301
Small-vessel occlusion	6.8	(4.8)	6.2	(6.2)	0.406
Other determined etiology	13.3	(14.7)	14.7	(12.5)	0.757
Undetermined etiology	10.5	(11)	10	(11)	0.597
Two or more causes identified	11.1	(9.4)	13.2	(12.8)	0.719
Negative evaluation	10.6	(7.8)	9.8	(10.4)	0.413
Incomplete evaluation	9.3	(15.4)	7.7	(11.9)	0.564
**Functional outcome on discharge**					
mRS 0–1, *n* (%)	245	(40.4)	242	(39.6)	0.788
mRS 2–3, *n* (%)	176	(29)	200	(32.7)	0.158
mRS 4–5, *n* (%)	85	(14)	90	(14.7)	0.718
In-hospital mortality, *n* (%)	100	(16.5)	78	(12.8)	0.067

AF, atrial fibrillation; SD, standard deviation; CS, cardioembolic stroke; PACS, partial anterior circulation stroke; POCS, posterior circulation syndrome; LACS, lacunar stroke; TACS, total anterior circulation stroke; NIHSS, National Institute of Health Stroke Scale; mRS, modified ranking score; DTI, door-to-imaging; DTN, door-to-needle; DTP, door-to-puncture; IQR, interquartile range.

The use of the gray shade emphasizes the distinct segments of the table, presenting the data in a more instructional and enjoyable manner for reading. In the last column to the right, the gray shade highlights the results that were statistically significant (p < 0.005).

The IV and MT rates were similar, although a higher absolute number of procedures occurred during the pandemic. The time intervals between hospital admission and IV/MT were also similar in both periods (Table 1).

The average in-hospital stay during the pandemic for almost all stroke patients was similar to the prior year. Only patients with cardioembolic stroke (CS) due to AF had a significantly shorter in-hospital stay (Table 1).

All patients were subjected to the same investigation protocol. Nevertheless, some exams were performed more frequently during the pandemic, such as CT (*p* = 0.025), TCD (*p* < 0.001), X-ray (*p* < 0.001), and TTE (*p* = 0.001). Conversely, MRI was performed less frequently compared to the previous year (*p* = 0.016) (Table 1).

There was no increase in in-hospital mortality during the pandemic, just a non-significant trend of reduction (*p* = 0.067) (Table 1).

## 4. Discussion

Sweeping restrictions were imposed in Joinville when the pandemic hit, including in healthcare. Primary Care Centers were reduced by one-third, and transformed into COVID-19 centers. Part of the clinical staff was directed to emergency demand. Home visits were suspended, and outpatient care became restricted to spontaneous demand. For patients with suspected or prior stroke, no alternative recommendation was made in the beginning. Every hospital had its structure, staff and space partially dedicated to treat patients with severe COVID-19. The referral hospital for stroke had its dedicated beds cut by 20% immediately. This initial impact justified an important reduction in admissions for stroke, particularly mild stroke, and TIA (11).

Despite this immediate impact, the present study showed a similar and even better stroke care system 1 year after the COVID-19 outbreak.

We observed similar demographic and clinical profiles. The gender distribution and mean age did not differ from the previous year. Similar data were published in a systematic review and by the National Registry of Stroke in the United States (29, 30). These data did not confirm the initial impression suggested by several reported cases of ischemic stroke associated with COVID-19 in younger patients and even children (1–6, 31–33). The prevalence of comorbidities and risk factors in patients with TIA and stroke did not change after the pandemic onset. Hypertension and a sedentary lifestyle were the most frequent risk factors, reaching 70% of cases, that confirmed previous classic findings in the literature (34).

Previous observational studies have pointed to important pandemic effects on stroke hospitalizations. A 28–40% reduction in admissions for all stroke, 19.1% reduction in hospitalizations for ischemic stroke, 17.1% reduction in large vessels ischemic strokes, 11.5% reduction for hemorrhagic stroke and 22.5% reduction for subarachnoid hemorrhage has been reported during the early 4 months (12–16). Despite these studies, our data revealed no significant variation between admissions for various stroke subtypes during the pandemic and the preceding year. Only a significant 32.8% decrease in the number of TIAs was observed. These results partially support the JOINVASC data that were previously published and showed a 41.2% decreased number of hospitalizations for mild stroke and TIA during the first 2 months of the pandemic (11). Our longer period of observation and a transitory impact of the pandemic on stroke dynamics can explain these findings. We also demonstrate that people with mild and especially temporary symptoms did not seek medical attention, maybe due to fear of contracting COVID-19 in crowded emergency rooms.

Previous studies have reported a higher severity in cases of stroke associated with COVID-19 (29, 35–37). The present study showed similar stroke severity before and after the pandemic's onset, probably due to the relatively low incidence of COVID-19 in stroke patients (5.06%). The incidence of COVID-19 in stroke patients admitted to hospitals worldwide is about 3.31% in the six continents, peaking at 8.93% in South America (17).

Regarding the etiological mechanism of stroke, the undetermined etiology with negative evaluation (cryptogenic) was similar and dominant in both periods in our cohort. The high prevalence of cryptogenic strokes has already been demonstrated in patients with stroke and COVID-19, with cryptogenic etiology ranging from 50 to 65% of cases (37, 38). In a meta-analysis, including almost 70,000 patients, cryptogenic ischemic stroke was more common in hospitalized patients with COVID-19 infection than in non-infected historical controls (35). The low incidence of COVID-19 in stroke patients during the pandemic period can again explain this finding. The prevalence of stroke with undetermined etiology due to incomplete evaluation was also similar in both periods. A greater difficulty in completing an adequate investigation during the pandemic was not observed, despite protective and isolation measures, which often delay or even prevent the performance of some complementary tests.

The investigative protocol for stroke patients did not change in the pandemic period. However, CT, TCD, X-ray and TTE were performed more frequently, and the number of MRIs decreased. It is likely that a stricter safety protocol for MRI—a time-consuming examination performed in a restricted environment and requiring rigorous cleaning protocols—can explain this decrease. These findings partially confirmed previous studies that showed a reduction in tests for stroke diagnosis. According to a published study, the number of neuroimaging tests for stroke reduced by 39% in the first 2 weeks of the pandemic (9). Another study involving 20 American states and using artificial intelligence counted a 22.8% reduction in the performance of important neuroimaging tests for acute stroke (14). In contrast, the increased use of some tests (CT, TCD, X-ray, and TTE) indicates a proper etiological investigation even in adverse conditions, and can be explained by updating in-hospital acute stroke protocols as well as structured training programs for hospital staff.

Only individuals with cardioembolic stroke due to atrial fibrillation had their hospital stays shortened. In order to ensure that these patients received appropriate anticoagulation at home, the public home care service and the local hematology center collaborated to collect and transport blood samples. These strategic and structural adjustments made it possible for patients to leave the hospital on safe follow-up arrangements sooner.

Access to reperfusion therapies before and during the pandemic was similar. IV and MT rates did not decrease from those of the previous year. In fact, we observed a non-significant absolute increase during the pandemic. These results differ from previously published data, which showed a 12.7–25% reduction in the MT rate in the first 3 months, as well as a 13.2–27% reduction in the IV rate in the first months after the pandemic onset (13, 14, 16, 17). Similarly, door-to-needle and door-to-procedure intervals did not worsen during the pandemic, which is extremely important since these therapies reduce mortality and have a time-dependent efficacy.

The in-hospital mortality of stroke patients did not increase during the pandemic period, in contrast to the previously reported data showing an increase in-hospital mortality by 9% after 1 year (14, 16). We believe this unchanged mortality rate is largely the consequence of a strong and effectively implemented approach for managing the recent pandemic challenges, rather than just a low prevalence of COVID-19 patients in our sample.

The pandemic collateral effect has been strongly demonstrated worldwide regarding stroke care, but our research suggests a manageable burden in Joinville during its first year. Indeed, the adverse novel circumstances of the pandemic compelled immediate strategic action to avoid further collateral damage on stroke care in Joinville. Such actions included (1) optimization of safety protocols for health professionals; (2) review of screening protocols for early COVID-19 detection in suspected stroke patients; (3) updating in-hospital acute stroke protocols; (4) maintenance of early safe post-stroke rehabilitation; (5) promotion of educational public campaigns about stroke awareness; and (6) increase in consistent training programs for health professionals. All these initiatives involved a multidisciplinary team as well as civil associations dedicated to reviewing practical protocols and implementing new strategies to ensure important information accessibility for patients, their relatives, caregivers and society at large. Telemedicine, open and social media calls, TV programs, interviews, flyers and billboard campaigns were useful to strengthen the urgent take-home message of “*stroke-don't-stay-at-home”* in contrast to “*stay-at-home* policy.” Local statistics and published data (11) encouraged persons experiencing signs and symptoms of stroke to seek emergency care immediately, regardless of the infection risk. Patients with cardioembolic stroke and atrial fibrillation received special attention by home caring monitoring.

Telemedicine has played a critical role in providing stroke care during the COVID-19 pandemic (39). The pandemic accelerated the regulatory process of telemedicine in Brazil. The letter from the Federal Board of Medicine (No. 1756/2020) published on March 19, 2020 recognized the possibility and ethics of using telemedicine exceptionally while the battle to fight the contagion of COVID-19 lasts (40). On March 31, 2020, the National Congress approved the Bill No. 696/2020, which allows for the use of telemedicine during the declaration of a public health emergency of coronavirus, and the Telemedicine Law (Law No. 13,989/2020) was institutionalized on April 15, 2020 (41). This made it possible to conduct remote consultations on an emergency basis in healthcare services across the country, which is particularly relevant for stroke patients. In Joinville, the Municipal Department of Health implemented this remote mode of treatment in March 2020. Two channels were made available to the population: (1) “Web-Saúde,” which operates through the WhatsApp messaging app, and (2) “Ligue-Saúde” for telephone calls. In July 2020, the scope and capacity of remote treatment were expanded through a “Virtual Clinic,” creating six teams of doctors and nurses who operate at a central level, answering calls from all over the municipality. Following the recommendations of the Ministry of Health, remote treatment (telephone call, message, email) has been used in the following situations: (1) Guidance on the correct use of medication and therapeutic adherence, healthy eating, regular physical activity, stress control, and other self-care topics; (2) Availability of medication and supplies; (3) Clarification of doubts; (4) Guidance on access to medication and procedures; (5) Reevaluation of therapeutic plan and monitoring; (6) Scheduling of in-person treatment with safety. In April 2020, due to the national difficulty in issuing prescriptions electronically, the Regional Board of Pharmacy and the Regional Board of Medicine developed a platform of electronic files with the professional's digital signature. As a result, the use of this platform was included in the process. Thus, the strategies adopted were many and telemedicine has been one of the most promising answers to approaching neurological care in pandemic times. International experience has shown that it potentially reduces the danger of infection for both patients and healthcare workers, as well as helps save time, make better use of resources, prevent needless transports or exposure, and offer safe home office work for medical specialists (42, 43).

### 4.1. Study limitations

A limitation of this study is that all analyses were performed on a mixed sample of COVID-19 positive and negative patients, with mostly COVID-19 negative patients, and some results were compared to studies that included COVID-19-positive stroke patients. Because of the early difficulties implementing COVID-19 testing in Joinville and a stroke management protocol that included COVID testing only for symptomatic patients, the low incidence of COVID-19-positive cases in our sample is probably underestimated. However, the pandemic's “collateral effect” on the local healthcare system may have an impact on both COVID-19 positive and negative patients.

Joinville has a privileged position as the first city in Brazil with a Public Stroke Unit, founded in 1997, that served as a great stimulus for improving local stroke care through to the current date. The ongoing efforts made the JOINVASC initiative recently recognized as the best Community Stroke Program in the world, winning the VBHC (Value Based Healthcare) 2021 prize and considered a model for other similar initiatives inside the country and abroad (44, 45). In other words, it is questionable whether the Joinville setting is indicative of most Brazilian contexts. Despite the great advances in stroke care in recent years, access to diagnosis and treatment of stroke remains highly heterogeneous even in the same country (46). Every city or region has its own health system context, COVID-19 infection curves with different behaviors, some have used distinct and even more radical strategies to contain the pandemic (such as “lockdown”). The results reported here from Joinville do not necessarily reflect the success (or otherwise) of strategies elsewhere in Brazil.

It is also important to note that Joinville has experienced an increase in COVID-19 vaccination rates over the course of our study (23). Since our study lacked these vaccine-related data, it is not possible to rule out a potential effect on reducing the risk of COVID infection-related complications and even strokes.

There were no records of any patient presenting with Cerebral Venous Thrombosis (CVT) in our study. Typical symptoms (headache, for example) and confirmed diagnosis of CVT (without ischemic or hemorrhagic complications) were not considered inclusion criteria. Although the JOINVASC registry is a database submitted to systematic quality control and weekly review by an experienced team, it is possible some cases of CVT diagnosis had been counted as ischemic stroke, intraparenchymal hemorrhage or even SAH. Similarly, some hemorrhagic stroke may have been a hemorrhagic transformation of a previous ischemic stroke indeed. Even with these possible lacunae, any possible change in our findings would have been relatively minor. According to available literature, such CVT cases represent only 0.08% of all cases of stroke and COVID-19, and hemorrhagic transformation represent only 0.7% of all ischemic strokes (47, 48).

## 5. Conclusions

Our study provides important real population-based data for understanding the dynamics of the COVID-19 pandemic and its consequences on stroke care in Joinville, Brazil. Statistics collected throughout the course of the first year following the start of the pandemic showed that TIA/stroke patients were neither younger nor presented more severe pathology on admission. TIA incidence decreased, and hospital stay was not longer, but shorter for patients with cardioembolic stroke and atrial fibrillation indeed. In the pandemic, we identified fewer MRI scans and more CT scans, TCD, X-ray, TTE. The access to reperfusion therapies and in-hospital mortality did not worsen. Finally, in-hospital stroke mortality did not increase during the pandemic.

We believe our findings reflect not only a low incidence of COVID-19 in stroke patients, but mainly an effective response of the local stroke care system. This study provides strong evidence that interdisciplinary initiatives, structured and well-developed services tend not to suffer such negative impacts under adverse external conditions, during the COVID-19 pandemic, even with scant resources.

## Data availability statement

The original contributions presented in the study are included in the article/supplementary material, further inquiries can be directed to the corresponding author.

## Author contributions

FR, CM, ML, and VZ contributed to conception and design of the study. JS and VN organized the database. FR performed the statistical analysis and wrote the first draft of the manuscript. All authors contributed to manuscript revision, read, and approved the submitted version.
